# *De Novo* Transcriptome Sequencing of* Olea europaea* L. to Identify Genes Involved in the Development of the Pollen Tube

**DOI:** 10.1155/2016/4305252

**Published:** 2016-02-21

**Authors:** Domenico Iaria, Adriana Chiappetta, Innocenzo Muzzalupo

**Affiliations:** ^1^Consiglio per la Ricerca in Agricoltura e l'Analisi dell'Economia Agraria (CREA), Centro di Ricerca per l'Olivicoltura e l'Industria Olearia (OLI), 87036 Rende, Italy; ^2^Università della Calabria, Dipartimento di Biologia, Ecologia e Scienze della Terra, Ponte Pietro Bucci, 87036 Arcavacata di Rende, Italy; ^3^Dipartimento di Farmacia e Scienze della Salute e della Nutrizione, Università della Calabria, Polifunzionale, Arcavacata, 87036 Rende, Italy

## Abstract

In olive (*Olea europaea* L.), the processes controlling self-incompatibility are still unclear and the molecular basis underlying this process are still not fully characterized. In order to determine compatibility relationships, using next-generation sequencing techniques and a* de novo* transcriptome assembly strategy, we show that pollen tubes from different olive plants, grown* in vitro* in a medium containing its own pistil and in combination pollen/pistil from self-sterile and self-fertile cultivars, have a distinct gene expression profile and many of the differentially expressed sequences between the samples fall within gene families involved in the development of the pollen tube, such as lipase, carboxylesterase, pectinesterase, pectin methylesterase, and callose synthase. Moreover, different genes involved in signal transduction, transcription, and growth are overrepresented. The analysis also allowed us to identify members in actin and actin depolymerization factor and fibrin gene family and member of the Ca^2+^ binding gene family related to the development and polarization of pollen apical tip. The whole transcriptomic analysis, through the identification of the differentially expressed transcripts set and an extended functional annotation analysis, will lead to a better understanding of the mechanisms of pollen germination and pollen tube growth in the olive.

## 1. Introduction

Fertilization in angiosperm plants is a complex process that includes several steps that may vary among species. It requires the successful transfer of the male gametes from the pollen grain through the pistil by means of the protrusion of a tubular gateway that grows down the style toward the embryo sac, the pollen tube. Upon germination, as the tip of the pollen tube extends, new cell wall material is continually deposited to maintain the integrity of the wall [1]. However, the success of pollination cannot disregard the complex set of interactions between the pollen grain and stigmatic surface [2]; depending on the species and the breeding period, the engraftment of the pollen grain may be mediated by the stickiness and surface tension of the stigmatic secretion [3] or through the mutual recognition of pollen coat and the proteinaceous pellicle of the stigma. The cytoskeleton, cell wall, and secretory dynamics are some of the fundamental features identified as crucial, but whose role has not yet been completely elucidated [4–6].

The pollen tube wall, which comprises an outer fibrillar layer, is mainly composed of pectin, hemicellulose, and cellulose, as well as a second, inner layer of callose. The callose lining is absent in the pollen tube tip. It is thought that pectins are polymerized and esterified within the Golgi and then transported to the growing wall by secretory vesicles. Pectins are then deesterified and cross-linked by Ca^2+^, resulting in a rigid framework that provides support for the growing tube. At the extreme tip of the pollen tube is the “clear zone.” The identities and activities of components present in this zone have not been fully established, and at present these remain controversial topics [7].

After germination, pollen tubes must grow directly within the extracellular matrix of the stigma tissue, an environment wealthy in polysaccharides, free sugars, proteins, glycoproteins, proteoglycans, lipids, and phenolic compounds [8], and later penetrate the transmitting tissue of pistil. A mature pollen grain, during its development, contains numerous enzymes, many of which are released upon contact with the stigmatic surface [9, 10]; in this context the balance established between the different enzymatic activities is important in promoting the success of the protrusion and penetration of the pollen tube: lipase, carboxylesterase, pectinesterase, pectin methylesterase, and pectinesterase inhibitor, for instance, participate in breaking down the polymers of cutin in the stigma cuticle and in regulating pollen tube wall dynamics in pistil tissues. In particular, the catalytic triad “Ser 153, Tyr167, and Lys171 (S-D-H),” at the active carboxylesterase site are required for pollen tube penetration of the stigma; callose synthase and cell wall glucanase regenerate the inner callose layer during tube wall remodelling; actin, actin depolymerization factor, and fibrin are important in remodelling in the apical and subapical regions of pollen tubes, both important aspects for rapid tip growth process; calcium binding protein and calmodulin binding protein maintain the tip-focused calcium gradient and modulate the distribution/transformation of pectins during pollen tube growth; apoplastic invertase, hexose transporter, and polygalacturonase are essential to import carbohydrates in the form of monosaccharides, used for callose plug formation [7].

Using modern next-generation sequencing (NGS) techniques, through Illumina RNA-Seq approaches, we have chosen to analyse the transcriptome dynamics of olive pollen tubes; reconstruction was performed together with a full-expression analysis, between samples obtained from different combination of pollen/pistil collected from self-sterile and self-fertile cultivars for a* de novo* transcriptome, in order to determine compatibility relationships between different cultivars and to identify differentially expressed gene sequences falling within gene families already described above and involved in pollen tube development.

## 2. Materials and Methods

### 2.1. Plant Materials

Pollen and pistil sampled from Nocellara del Belice and Nera di Gonnos cultivars are grown* in vitro* for three parallel trials in mediums containing a combination of pollen/pistil from self-sterile (“Nocellara del Belice”) and self-fertile (“Nera di Gonnos”) cultivars and a medium containing a cross-pollination trial between pollen grain and pistil from “Nocellara del Belice” and “Nera di Gonnos,” respectively.

### 2.2. RNA-Seq Library Preparation and Sequencing

In order to obtain a general overview of the transcripts and metabolic pathways during pollen tube growth and to avoid cross contamination from nonhomogeneous tissue separation, sample pooling strategy was used here [11, 12]. Pooling reduces variability by minimising individual variation and represents an alternative approach to biological replicates in experiments where interest does not focus on the individual but rather on characteristics of the population (e.g., common changes in expression patterns) [13, 14].

Total RNA was extracted, at each sampling, from the excised pistil and pollen grain using the RNeasy Plant Mini kit (Qiagen) according to the manufacturer's instructions. Each RNA sample was subjected to DNase digestion (DNase I, Roche) to remove any remaining DNA and pooled equally, as previously described [15]. RNA was quantified by the NanoDrop Spectrophotometer ND-2000 and quality was checked by electrophoresis (28S rRNA/18S rRNA ratios). Samples with a concentration of ≥ 400 ng/*μ*L, OD260/280 = 1.8~2.2, RNA 28S : 18S ≥ 1.0, and RNA Integrity Number (RIN) ≥ 7.0 were used for cDNA library preparation.

Standard RNA-Seq library preparation and sequencing via Illumina HiSeq TM 2000 were carried out by Technology Services of the Institute of Applied Genomics (IGA, Udine, Italy); for each sample a single-end (SE) sequencing cDNA library was constructed with a fragment length range of 50 bp. Each of the libraries was performed using two replicates consisting of a separate pool of 10 homogeneous samples.

### 2.3. RNA-Seq Data Filter and* De Novo* Assembly by Trinity

The raw Fastq “reads” (NCBI PDA/bioProject PRJNA308210, bioSample accession numbers: SAMN04388479, SAMN04388480, and SAMN04388481, Table 1) were analysed and filtered, respectively, with FastQC and Fastx Toolkit to obtain high quality* de novo* transcriptome sequence data. Each sequence set was filtered with these criteria: first, the read containing the sequencing adaptor was removed; second, the reads with unknown nucleotides comprising more than 5% were removed; and third, low-quality reads with ambiguous sequence “N” were trimmed and discarded. Subsequently, without a reference genome a* de novo* assembly of the clean reads into transcripts was performed using Trinity, a novel method for the efficient and robust* de novo* reconstruction of transcriptomes from RNA-Seq data [11, 16–23].

Trinity was run via script using 128 GB of ram, 12 cpu thread, and a minimum assembled contig length to report set to 300 bp.

Trinity sequentially combines Inchworm, Chrysalis, and Butterfly modules to process large RNA-Seq reads data, partitioning the sequence data into many individual de Bruijn graphs, representing transcriptional complexity at a given gene or locus [16, 23].

### 2.4. Analysis of Transcript Assembly

For nonmodel organisms one metric for evaluating the assembly quality is to examine the number of transcripts that appear to be full-length or nearly full-length if compared to a closely related organism to examine full-length coverage. In this context a more general analysis was performed aligning the assembled transcripts against all known plant proteins determining the number of unique top matching proteins that are aligned in 70–100% range of their length by full-length transcript analysis [16]. Therefore, a blastable database has been created to perform a local BLASTX search where only the single best matching Trinity transcript is outputted for each top matching entry.

To validate our* de novo* assembly read remapping was conducted using bowtie2 [24]; for each data set a bowtie2 index was created, and then the number of reads that map our transcriptome was counted (Table 1).

### 2.5. Abundance Estimation and Differentially Expressed Trinity Transcripts

For abundance estimation of transcriptome assemblies RSEM software was used [25]. RSEM is a package for estimating gene and isoform expression levels from RNA-Seq data. Moreover, Trinity currently supports the use of bioconductor tools (EdgeR) to compute differential expression analysis in the assembled transcriptome [16, 23, 26, 27]. In order to identify statistically significant differences in transcript expression between samples, it is necessary to consider the number of reads/transcripts, the depth of sequencing, the length of the transcripts (longer transcripts generate more fragment reads), and the expression level of the transcripts. Expression values normalized for each of these factors are measured in FPKM (fragments per feature kilo base per million reads mapped) [28] and to make a comparison across multiple samples and replicates. Trinity supports the use of TMM (trimmed mean of *M* values) normalization [29, 30], to account for differences in the mass composition of the RNA-Seq samples, which does not change the fragment count data but instead provides a scaling parameter that yields an effective library size (total mappable reads) for each sample. This effective library size is then used in the FPKM calculations.

### 2.6. Annotation

To compute overexpressed Gene Ontology (GO) terms in our transcriptome, we used BLASTX 2.2.26+, BLOSUM62 similarity matrix, and Blast2GO database version August 2011 [31, 32]. The definition of each GO term is determined by the GO Consortium, http://www.geneontology.org, and can be found using the EMBL European Bioinformatics Institute QuickGO, http://www.ebi.ac.uk/QuickGO, or the Gene Ontology Normal Usage Tracking System, GONUTS. Pathway assignments were determined following the Kyoto Encyclopedia of Genes and Genomes pathway database [28, 33] using BLASTX with an *E*-value threshold of 1.0^−5^. MapMan (http://mapman.gabipd.org/) analysis was conducted using our DE transcripts rearranged as input experimental dataset, to assign MapMan “BINs” to DNA sequences [34, 35]. The output was used as a mapping file for data visualization.

## 3. Results and Discussion

### 3.1. RNA-Seq Library Sequencing and* De Novo* Transcriptome Assembly by Trinity

Starting from three Illumina RNA-Seq libraries, corresponding to different pollen/pistil combinations from self-sterile and self-fertile olive cultivars, “Nocellara del Belice” and “Nera di Gonnos,” respectively, 154,525,512 raw reads were generated from 50 bp insert library. A total of 140,357,776 high quality SE reads were identified and used for transcriptome assembly through Trinity software. Using the 25-mer in Trinity, which is recommended by its authors [16, 23], as well as a minimum assembled contig length set to 300 bp, we found 15,317 transcripts; total used reads and total assembled transcripts and N50 statistics for each sample are indicated in Table 1.

### 3.2. Differential Expression Analysis

To estimate differential gene expression between each pollen/pistil combination a single assembly based on combining all reads across all samples as inputs was generated, to avoid difficulty in comparing results across the different samples, due to differences in assembled transcript lengths and contiguity. Then, reads were aligned separately back to the single assembly in order to identify the number of differentially expressed (DE) transcripts having a significant false discovery rate (FDR) value of at most 0.001 and at least fourfold difference in expression values according to the Trinity protocol.

**Figure 1:**
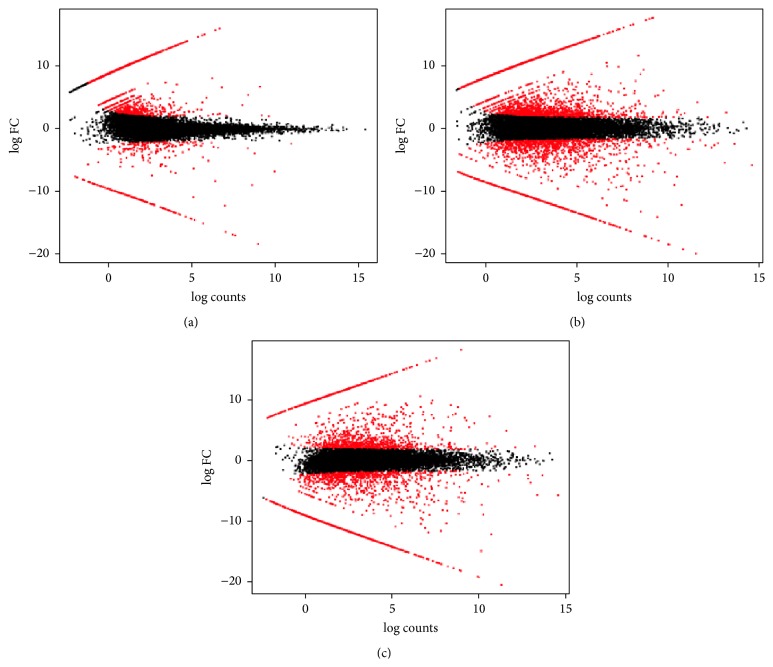
Pairwise comparisons of transcript abundance from self-pollination in self-sterile cultivar versus cross-pollinated sample (a), from self-pollination in self-fertile cultivar versus cross-pollinated sample (b), and in between self-pollination in self-sterile cultivar and self-pollination in self-fertile cultivars (c). *MA* plot for DE analysis by EdgeR: for each gene, the log_2_(fold change) (log_2_(sample (a)/sample (b))) between the two samples is plotted (*A*, *y*-axis) against the gene's log_2_(average expression) in the two samples (*M*, *x*-axis).

It was possible to identify 2802 DE transcripts; fold change and statistical significance values were also estimated (Figure 1).

Trinity facilitates analysis of RNA-Seq data, including scripts for extracting transcripts that are above some statistical significance (FDR threshold) and fold change in expression. To examine expression across multiple samples, the FPKM expression values across samples were normalized, which will account for differences in RNA composition, and afterwards TMM normalization generates a matrix of normalized FPKM values across all samples.

These adjusted library sizes are used to recompute the FPKM expression values. Although the raw fragment counts are used for differential expression analysis, the normalized FPKM values are used below in examining profiles of expression across different samples; each DE set of transcripts was displayed as *MA* plots (where *M* is log ratios and *A* is mean values) (Figures 1(a)–1(c)).

### 3.3. Functional Annotation of Transcript Sets

**Figure 2:**
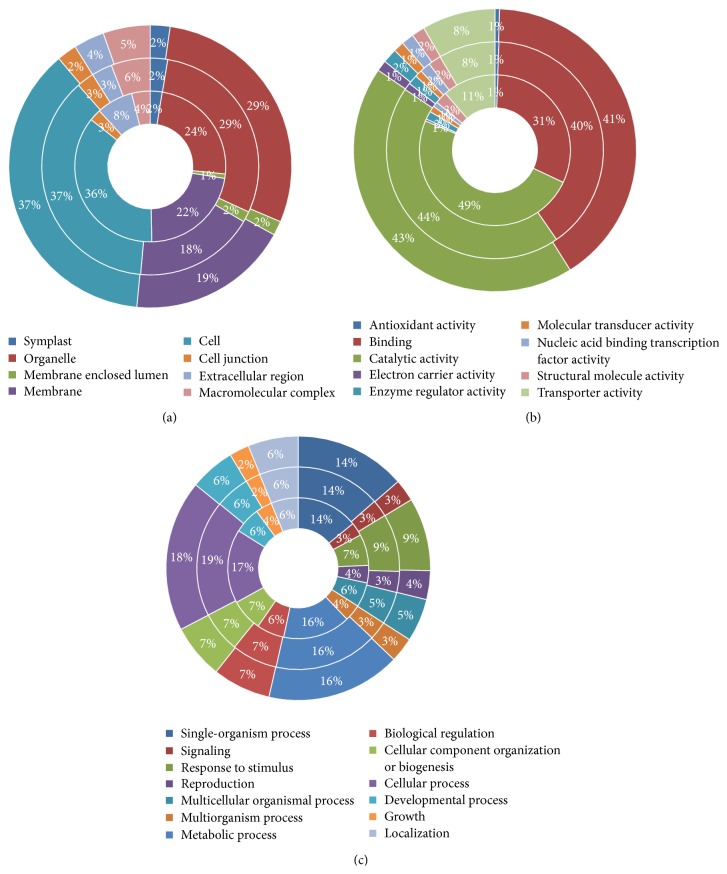
Distribution of ontological categories (level 2 GO terms) in self-sterile (inner chart), cross-pollinated (middle chart), and self-fertile cvs (outer chart) DE transcripts according to cellular component (a), molecular function (b), and biological process (c). The percentage of the transcripts within each class is reported.

The* in silico* analysis of the entire sets of DE transcripts, conducted through querying databases of genes and proteins (NCBI, ExPASy, and InterProScan) and the functional annotation software Blast2GO, has allowed each sequence to be traced back to the gene family and to be annotated according to the terms of the three main Gene Ontology vocabularies (image data not shown). Unexpectedly, a fairly overlapped distribution of GO terms is observed (Figures 2(a)–2(c)); in particular, the most represented ontological categories at a cellular component level in our gene sets are between sample cell, organelle, and membrane. Molecular function categories are strongly represented by terms related to catalytic activity, binding, and transporter activity. Finally, more than ten categories were identified at the biological process level with metabolic and cellular processes among the groups most represented, highlighting the intense and complex metabolic and regulatory activities during fruit maturation.

**Figure 3:**
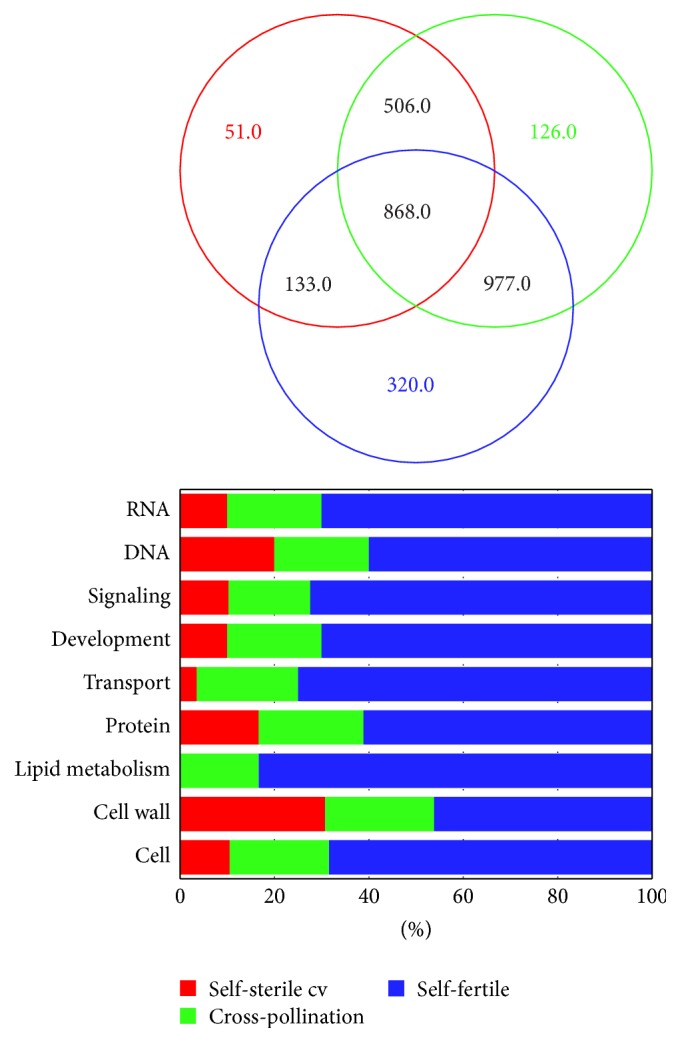
Venn diagrams and clustering of DE transcripts in their relationship with the analysed samples. For each sample, the categories most represented are displayed as stacked bar graph.

In order to trace back to the pathways, which are more closely involved in pollen tube growth between samples, the whole sets were examined through the Kyoto Encyclopedia of Genes and Genomes (KEGG), focusing attention on the DE transcripts, and implemented in MapMan, to focus the main DE gene via BIN codes functional classification (Figure 3). The visualization of the DE set reveals that only 868 transcripts are shared between samples, with cell wall, protein, and transport among the most representative functional classes. In particular, of the whole DE set, it should be emphasized that 51 transcripts are expressed only during pollen tube growth in self-sterile cultivar, with BIN related to protein degradation categories, while 126 and 320 transcripts are present in cross-pollination and self-fertile experiments, in which cell wall degradation, RNA processing, lipid metabolism, transport, and signalling are the most consistent functional BINs. In this context, a comprehensive view of the DE transcripts distributed among the samples allows for the highlighting in Figure S1 (in Supplementary Material available online at http://dx.doi.org/10.1155/2016/4305252) of the most representative functional classes that resulted from the VENN diagram.

**Figure 4:**
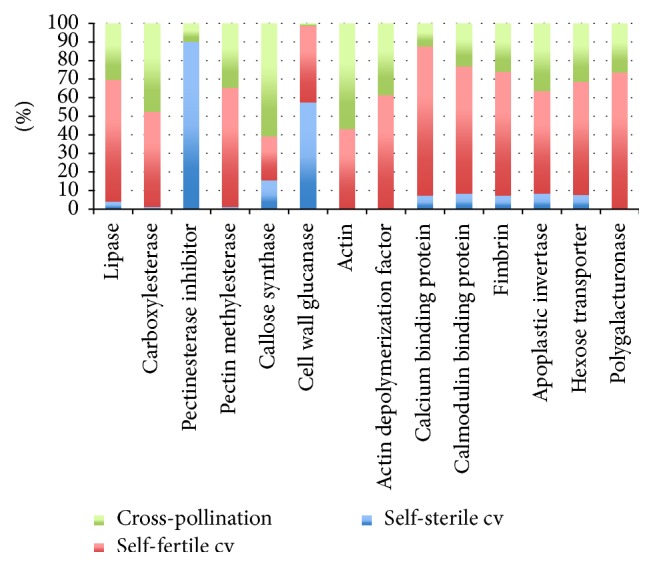
Transcript abundance between samples. Each data is displayed as a stacked bar. Transcript expression levels were taken from the complete FPKM normalized plots that were identified as differentially expressed.

In those circumstances and within these groups, by deepening the different transcripts mainly involved in signal transduction, transcription, and cytoskeleton dynamics, which are overrepresented in particular in the self-fertile cultivar and are directly correlated with tube pollen germination, such as lipase, carboxylesterase, pectinesterase, pectin methylesterase, and callose synthase (Figure 4), the analysis also allowed us to identify members in actin, actin depolymerization factor, and fibrin gene family and member of the Ca^+2^ binding gene family, related to development and polarization of pollen apical tip, as well as to inhibitors (pectinesterase inhibitor) regulating pollen tube wall dynamics in pistil tissues (Table S1). The expression abundance of our candidate transcripts chosen as markers in pollen tube development seems to be so under the control of a delicate balance between the different gene families (Figure 4). In particular, these transcripts follow a different trend if we compare self-fertile/self-sterile cultivars and cross-pollination sample, resulting as more expressed, as would be expected, in the self-fertile cultivar if compared to the other samples.

The whole transcriptomic analysis, through the identification of differentially expressed transcripts together with an extended functional annotation, seeks to make a valuable contribution to better understand the mechanisms involved in pollen germination and pollen tube growth and the signals that regulate the interaction between a pollen tube and pistil structure in its journey to generate tools for breeders in their quest to break species barriers and produce novel hybrids.

## Supplementary Material

Figure S1: The in silico analysis of the entire differentially expressed set, conducted by querying gene and protein database (NCBI, ExPASy, InterProScan) and the functional annotation, conducted by Blast2GO, has allowed for each assembled differentially expressed transcript to be traced back to the gene family and to the ontological category to which they belong. The entire set was then clustered in relation to cellular component, molecular function and biological process.
Table S1: List of selected genes identified as marker of pollen tube development. Best matches identified in the ReprOlive and Tair database are reported.

## Figures and Tables

**Table 1:** Total used reads and total assembled transcripts and statistics for analysed samples.

Sample	Accession	Reads	Transcripts	Contig N50	Mapped reads
Self-pollination in self-sterile cv	SAMN04388479	25903277	479	474	82.58%
Self-pollination in self-fertile cv	SAMN04388480	40278164	4800	551	85.67%
Cross-pollination	SAMN04388481	74176335	10038	508	86.85%
