# Supplementary material for: De Novo Transcriptome Sequencing of Olea europaea L. to Identify Genes Involved in the Development of the Pollen Tube
Source: ScientificWorldJournal. 2016 Feb 21;2016:4305252. doi: 10.1155/2016/4305252 (PMC4779530; doi:10.1155/2016/4305252)
Supplement: Supplementary file 1 — Figure S1: The in silico analysis of the entire differentially expressed set, conducted by querying gene and protein database (NCBI, ExPASy, InterProScan) and the functional annotation, conducted by Blast2GO, has allowed for each assembled differentially expressed transcript to be traced back to the gene family and to the ontological category to which they belong. The entire set was then clustered in relation to cellular component, molecular function and biological process. Table S1: List of selected genes identified as marker of pollen tube development. Best matches identified in the ReprOlive and Tair database are reported. [file 4305252.f1.docx]

**Fig S1**


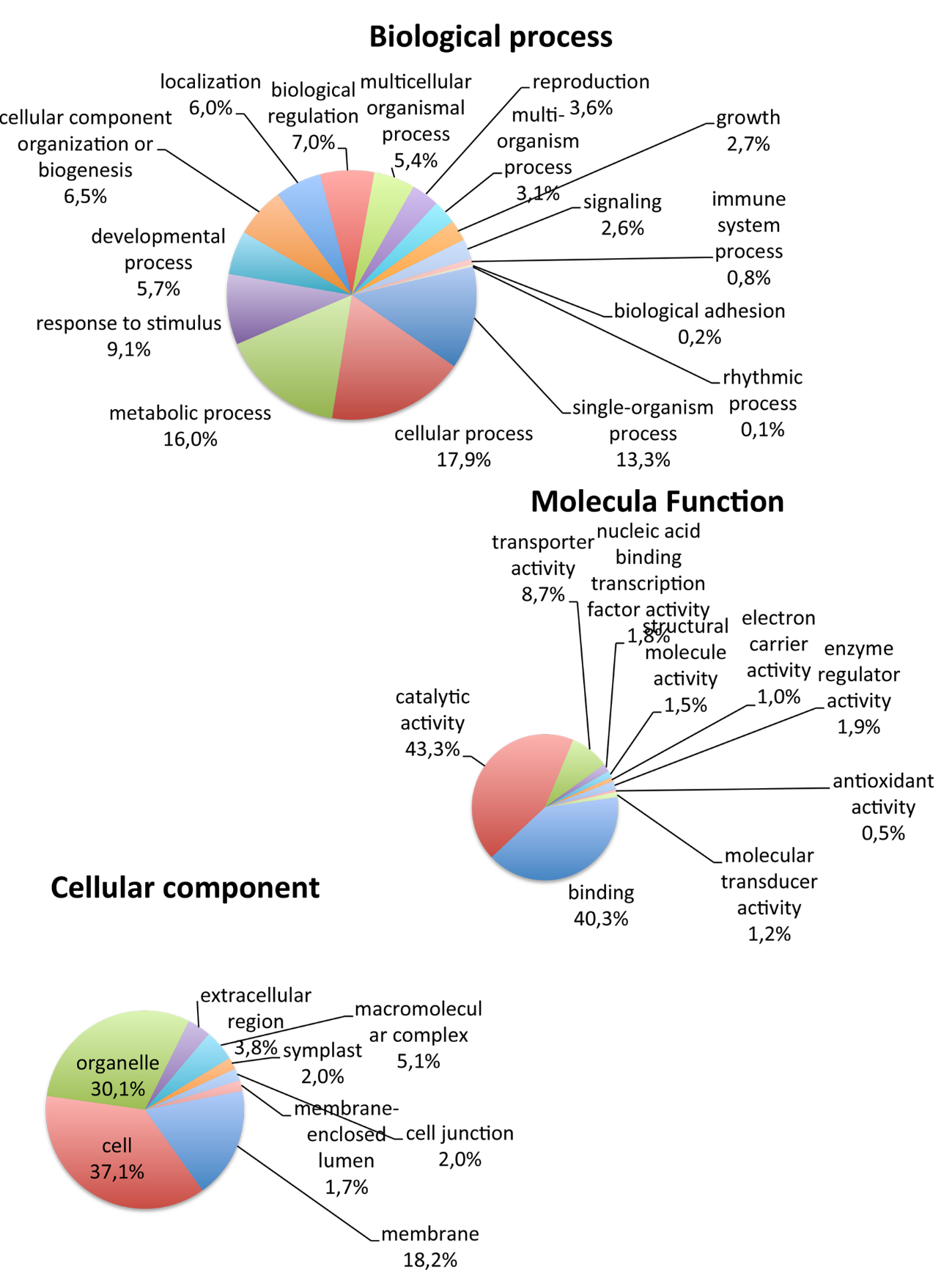


**Table S1**

| Name | Description | Length | ReprOlive* | Identities | TAIR** | Identities |
| --- | --- | --- | --- | --- | --- | --- |
| comp11541 _seq1 | Lipase | 1176 | rp11_olive_008037 | 99% | AT1G10740 | 78% |
| comp8782 _seq1 | Carboxylesterase | 304 | rp11_olive_049553 | 100% | AT2G45610 | 59% |
| comp374 _seq1 | Pectinesterase Inhibitor | 459 | rp11_olive_003895 | 100% | AT5G27870 | 48% |
| comp12963 _seq1 | Pectin Methylesterase | 673 | rp11_olive_027343 | 98% | AT5G07430 | 54% |
| comp358 _seq1 | Callose synthase | 411 | rp11_olive_000021 | 99% | AT2G13680 | 91% |
| comp9373 _seq1 | Cell wall glucanase | 333 | No hits found |  | NP_197539.1 | 69% |
| comp4481 _seq1 | Actin | 308 | rp11_olive_042755 | 100% | AT5G09810 | 83% |
| comp11811 _seq1 | Actin depolymerization factor | 590 | rp11_olive_032997 | 99% | AT4G25590 | 80% |
| comp11729 _seq1 | Calcium binding protein | 313 | rp11_olive_066925 | 99% | AT3G03430 | 75% |
| comp11371 _seq1 | Calmodulin binding protein | 406 | po11_olive_001341 | 99% | AT3G13600 | 53% |
| comp14223 _seq1 | Fimbrin | 629 | rp11_olive_002193 | 99% | AT5G35700 | 83% |
| comp209 _seq1 | Apoplastic invertase | 353 | rp11_olive_005301 | 100% | AT2G36190 | 66% |
| comp1872 _seq1 | Hexose transporter | 902 | po11_olive_000437 | 99% | AT3G05960 | 60% |
| comp13837 _seq1 | Polygalacturonase | 608 | rp11_olive_021027 | 100% | AT4G20050 | 65% |
| * http://reprolive.eez.csic.es  ** http://www.arabidopsis.org | | | | | | |
